# Expansion of perceived size of visual stimuli: Objects look wider than equivalent empty spaces

**DOI:** 10.1177/03010066251359214

**Published:** 2025-07-29

**Authors:** Algis Bertulis, Arunas Bielevicius

**Affiliations:** 230647 Lithuanian University of Health Sciences, Kaunas, Lithuania

**Keywords:** relative size, expansion phenomenon, object outlines, luminance contrast, colour contours, texture borders, perceptual grouping, and illusory contours

## Abstract

The study builds upon previous research on the perceived size of visual objects of various shapes compared to an empty spatial interval. In psychophysical experiments using the size-matching procedure, the effect of overestimating the relative size of an object (relative to an equivalent empty space) was consistently observed when testing visual objects, such as rectangles, circles, ellipses, rhombuses, and triangles, in both filled and empty formats. The strength of the illusion did not depend on whether the shapes were filled or not, but rather it varied with the shape itself. Objects with open contours, such as angles of different orientations and narrow stimuli like straight, tangled, defocused, and divided lines, all produced the expansion effect. The overestimation manifested during testing stimuli of various contour types, including spatial contrast of luminance, colour, and texture, as well as those determined by perceptual grouping and illusory outlines of Kanizsa and Oppel-Kundt versions. Finally, the expansion effect was found to be more pronounced with increasing length and height of the stimuli. The data supported the assumption that the object contour is the primary inducer of perceived size expansion and that the overestimation effect is a regular phenomenon rather than an incidental event.

## Introduction

The human visual system can determine relative sizes by comparing the distances between selected segments in a single object or many objects. However, misperceptions of relative size are highly probable. Two objects of the same physical size may not appear the same. The widely known and well-documented optical illusions of the Müller–Lyer and Oppel–Kundt types illustrate phenomena of overestimation and underestimation. According to the Müller–Lyer test (1889), a line flanked by outward-pointing wings seems to be more extended than a line of the same length flanked by inward-pointing wings. Judged separately, the line in the figure with outward wings was overestimated in comparison with the test line with no wings (expansion), and the line with inward wings was underestimated (contraction) in comparison with the test (Robinson, 1998). The Oppel–Kundt illusion (Wade et al., 2017) demonstrates that an area filled with a regular sequence of uniform stripes appears longer than an empty area of the same length, a phenomenon known as the expansion effect. When the Müller–Lyer wings were superimposed on an Oppel–Kundt stimulus, the two visual illusions were combined to balance each other (Bertulis Čerkelis et al., 2018; Bulatov et al., 2024), despite the different mechanisms of illusion origin. The expansion–contraction effect of the Müller–Lyer type is a rapid process, whereas the expansion of the Oppel–Kundt type is a slow process (Bertulis et al., 2014). The mechanism encoding the centroids of the flanking wings (Bulatov, 2017; Bulatov et al., 2011) is likely to be located at an earlier level, whereas the Oppel–Kundt mechanism of unexplained structure and functioning can be situated in the later levels of the visual pathways (Bertulis et al., 2014).

More data on perceived size expansion is known. Visual objects formed of line contours cause the expansion of perceived length, height, and, simultaneously, size (Makovski, 2017). Disconnected or missing boundaries provide more potent illusions than continuous or fully closed boundaries. The stimuli of a uniform block, contour of a rectangle, blurred contours, and grey or coloured patterns balanced the overestimation of the extension of the stripe sequence, the Oppel–Kundt figure (Bertulis et al., 2019). Moreover, the stimuli themselves caused the effect of overstating the length compared to the test distance. It is assumed that any visual object appears larger than it is during the size-matching procedure (Bertulis et al., 2019). The actual contours of a solid bar and the imaginary contours of a regular sequence of filling elements are suspected to be the main culprits for expansion development (Bertulis et al., 2014). A contour is the most informative part of an object (Loffler, 2008) that defines the shape and provides knowledge about the spatial proportions and distances between the outline parts.

This study focused on these assumptions and examined the expansion effect. New data were collected through psychophysical experiments with comparison procedures in which stimuli consisting of two parts – a reference object and a test space – were used. The experiment investigated whether the referential distance was perceived as longer, shorter, or equal to the test distance. Some aspects were considered: i) the influence of the shape of stimuli on the distance assessment, ii) the impact of different types of object outlines, iii) the influence of the stimulus size, iv) the meaning of the filled and empty stimulus areas, and v) the effects of contraction. The experiments yielded compelling results.

The object itself determines the sense of relative size. However, some other neural mechanisms functioning at different levels of the visual system contribute to the perception of the object (Coren & Girgus, 1978). Therefore, the perceived relative size is influenced by certain contextual features, such as the surroundings of the target object (Boswell et al., 2021; Obonai, 1954; Wundt, 1898), partial occlusion (Palmer et al., 2007), or motion (Ansbacher, 1944; Li et al., 2009; Mruczek et al., 2014), but these features were absent in the stimuli used in this study and they did not fall within the scope of this study.

## Methods

### Stimuli and Apparatus

**Figure 1.**
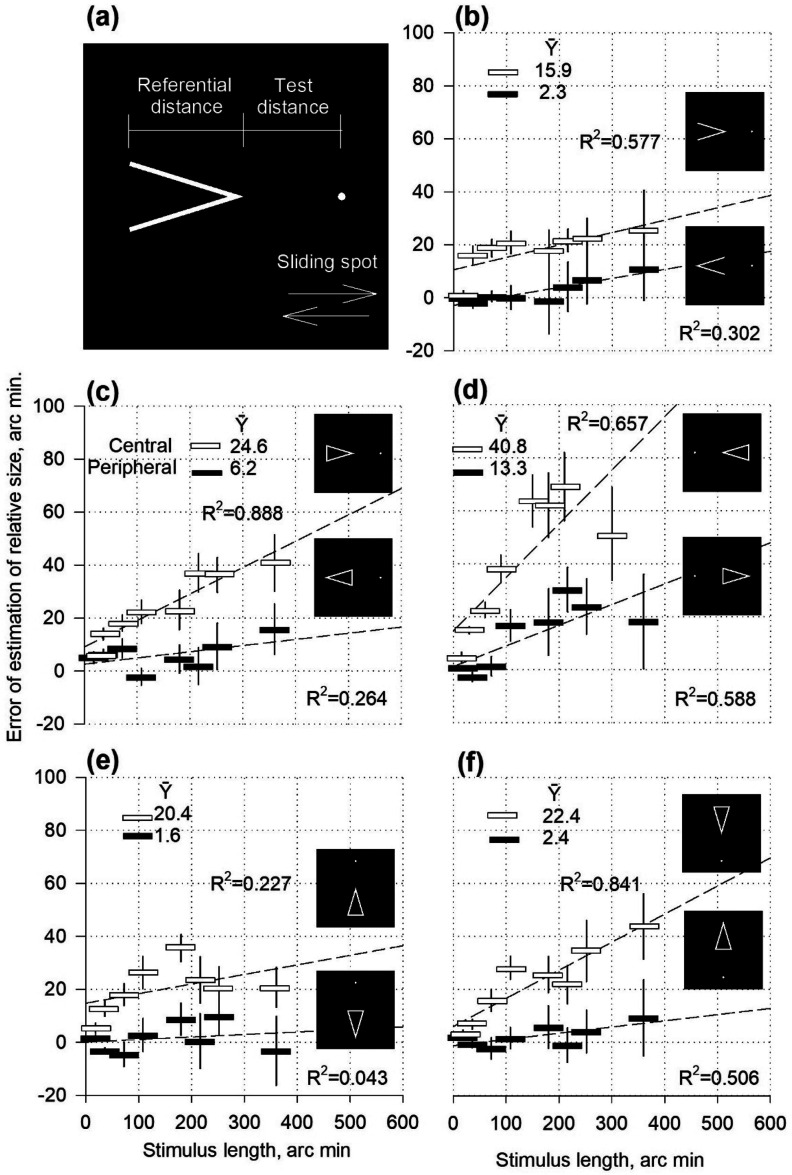
Estimation of the relative size of angles and triangles as functions of stimulus length or height.

*Note.* In (a), a facsimile of a horizontal two-part stimulus (explanations in Methods). In graphs (from b to f), experimental data are represented by white and black horizontal bars, indicating results for central and peripheral apex orientation stimuli, respectively. Vertical bars, confidence intervals. Dashes, regression lines. $\bar{Y}$ , mean values in arc min. Stimuli facsimiles flanking regression lines illustrate the referential objects on the left (b and c) and correct (d) side of the horizontally aligned two-part stimuli, or presented in the lower (e) or upper (f) part of the vertically oriented stimuli. Pilot experiments, subject A.

Bright stationary stimuli were presented against a dark background on a monitor screen. The stimulus patterns comprised referential and test parts aligned horizontally side by side (Figure 1) or at a certain distance apart (Figure 5(a), c_1_, and c_2_). In the referential section, 2D visual objects (e.g., geometric figures, such as angles) are presented (Figure 1(a)). The test distance (space) was limited to the object's apex tip and the spot (2.4 × 2.4 arc min in size, Figure 1a) or the vertical stripe (2.4 arc min in width) and the border of the texture (Figure 5(a)). In the pilot experiments, the reference objects (angles and triangles) were situated on the left (Figure 1(a) to (c)) or right sides (Figure 1(d)) of the horizontally aligned two-part stimulus. Vertically aligned stimuli were also tested (Figure 1(e) and (f)). The lower (Figure 1(e)) and upper positions (Figure 1(f)) were obtained. In the subsequent experiments, the left horizontal part was used as the reference. The initial distance of the test was randomised during the presentation of the stimuli and evenly distributed within 30% of the length of the referential stimulus.

**Figure 2.**
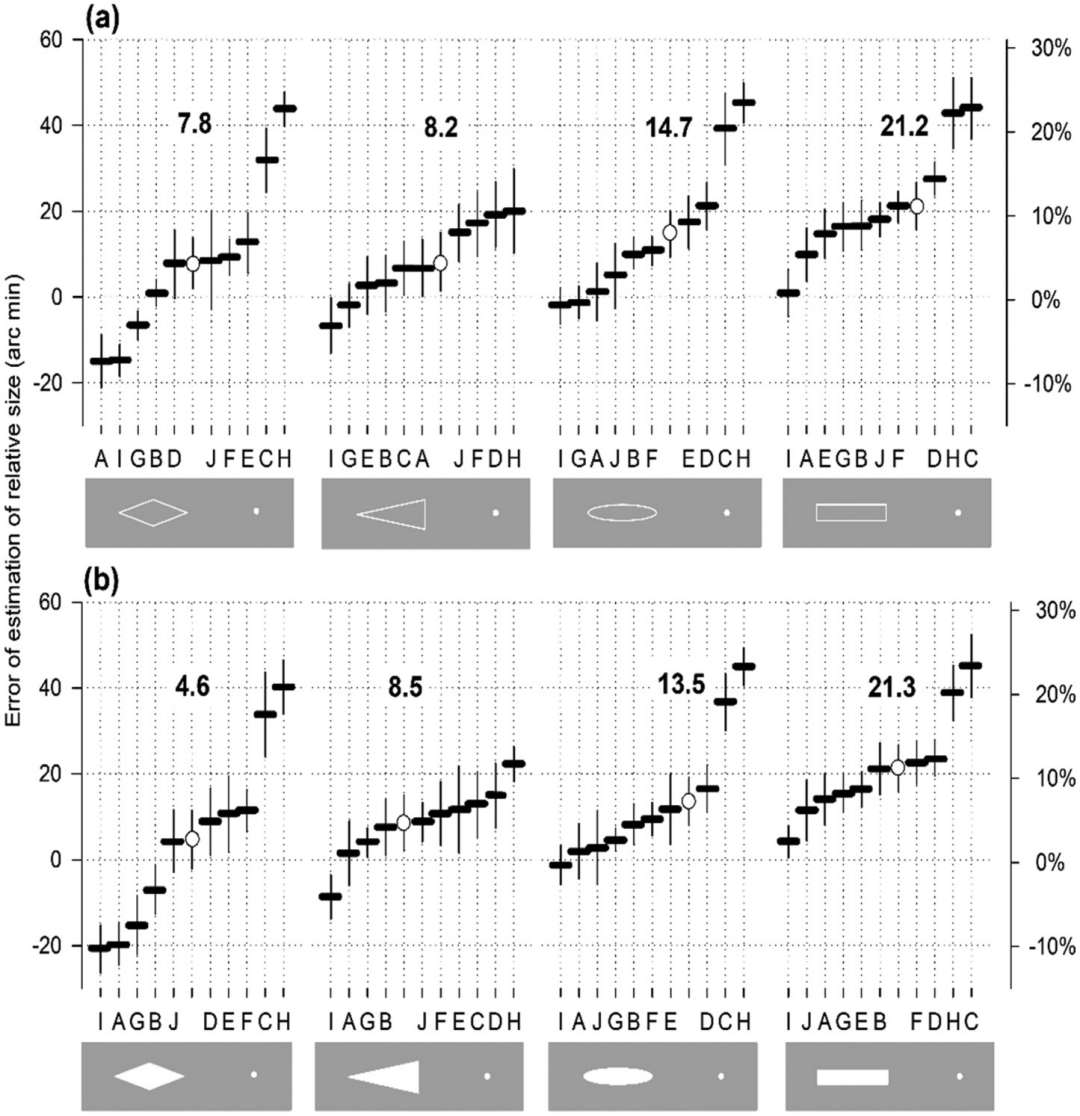
Estimation of relative length of stimuli differing in shape.

*Note.* Facsimiles of the stimuli formed by line contours are presented in (a) and filled shapes in (b). In graphs, an ascending order of the horizontal bars shows individual results of subjects: Capital letters above the corresponding stimuli. Circles and figures above, average values, arc min. Vertical bars, confidence intervals. The scale of percentages on the right represents the misestimation strength per unit of distance – spatial effectiveness.

The Results section provides a detailed description of the stimuli by group. Cambridge Research Systems VSG 2/3 drew and displayed the stimuli on EIZO T562 T, CRT (800 × 600 pixels), or OLED48C11LB (3840 × 2160 pixels) monitors. They were calibrated and gamma-corrected using a Cambridge Research Systems OptiCAL photometer. The Psychophysical Experiment Toolbox (Surkys et al., 2021), designed by the authors, is implemented within the MathWorks MATLAB software platform. It controls stimulus presentations, introduces changes according to the subjects’ commands, and records the subjects’ responses.

### Subjects

Thirty-eight volunteers, comprising 25 university students (25 women and four men; mean age, 20.7 years) and nine university teachers and employers (six women and three men; mean age, 49 years), participated in the experiments. All subjects had normal vision or wore their usual optical corrections. None of the students had practised any similar procedures and were naïve to the goals of the study. The lecturers were informed about the purpose and methods of the investigation. No differences were observed between the data obtained by the two participant groups. For example, the results of Lecturer B (Figure 2) fall in the middle of the students’ performance.

All subjects provided informed consent before the experiments, as outlined in the Declaration of Helsinki of 1964. The authors received written approval No. BE9-5 from the Kaunas Regional Biomedical Research Ethics Committee.

### Procedure

The experiments were conducted in a dark room. The subjects watched the monitor screen monocularly through an artificial pupil with a diameter of 3 mm. The distance between the screen and the subject's eye was either 100 or 150 cm, which caused the screen elements to correspond to 1.2 × 1.2 arc minutes or 0.5 × 0.5 arc minutes, respectively. The support of the forehead and chin limited the movement of the head. No instructions were provided regarding gaze fixation, and the observation time was unlimited. The method of Adjustments was used in the experiments. Randomising stimuli with different parameters in the presentation sequences reduced the bias in the judging criteria, which is an inherent characteristic of the method. The subjects were asked to adjust the test distance (by moving the lateral terminator, sliding spot (Figure 1(a)), or vertical stripe (Figure 5(a)) of the test part by one pixel at a time) to make it perceptually equal to the length or height (Figure 1(e) and (f)) of the referential stimulus. For example, subjects moved the stimulus spot to the left or right (see the stimulus facsimile in Figure 1(a)) or up and down (Figure 1(e) and (f)), and then fixed it in a position that determined the equality between the test distance and the length or height of the triangle. The errors made by the subjects were considered a measure of the magnitude of distortions in visual perception, that is, the strength of the overestimation or underestimation of relative size. The participants’ judgements were documented in angular minutes and percentages. Arc minutes refer to the total magnitude, and percentages show the spatial effectiveness of misestimates (magnitude per unit of distance). Participants were not provided with additional practice or knowledge of the results during the experimental sessions.

### Analysis

This study included data from 750 experimental runs. Seven to 11 stimuli were presented twice to an observer during each run. Five runs were performed on different days. Each subject was tested with each stimulus 10 times, and each data point analysis included the results of the 10 presentations.

Data were checked for normal distribution (Shapiro–Wilk) and equality of variance (Brown–Forsythe). Accordingly, analysis methods using MS Excel and SigmaPlot for Windows (Systat Software, Inc.) were employed. Data were analysed using descriptive statistical methods. Parametric tests were performed for the normally distributed variables. Student's *t*-test (one-sample *t*-test, independent samples *t*-test, and paired samples *t*-test) was used to compare the means between the two groups. An *F*-test (one-way ANOVA) was used to compare the means of three or more groups. Nonparametric methods were also employed. The Mann-Whitney U and Wilcoxon *Z*-tests were used as alternatives to the Student's *t*-test, while the Kruskal–Wallis H test was used as an alternative to the *F*-test (ANOVA).

## Results

In the pilot experiments, overestimation of length and height was demonstrated by testing asymmetric stimuli: angles and triangles (Figure 1). The magnitude of the overestimation gradually increased with the increasing length or height of the reference figure in all cases (b, c, d, e, and f in Figure 1). This effect was observed in all cases without exception, confirming the expansion phenomenon. The expansion can be attributed to the processing of contour-specific excitations (Bertulis et al., 2019).

The errors were larger for the central orientation of the reference figure (the apex of the figure pointing toward the stimulus centre) than for the peripheral orientation (the apex pointing toward the stimulus periphery). Angles (Figure 1(b)) of the central orientation gave a mean expansion value ( $\bar{Y}$ ) of 15.9 arc min. The peripheral orientation showed a mean error ( $\bar{Y}$ ) of only 2.3 arc min, which was six to seven times lower than the overestimation in the central orientation. The triangles behaved similarly. For horizontal triangles on the left side of the two-part stimulus (Figure 1(c)), the central orientation's mean error value ( $\bar{Y}$ ) was 24.6 arc min. Peripheral orientation was four times less, at 6.2 arc minutes. When the triangles were situated on the right side (Figure 1(d)), the central orientation prevailed over the peripheral again: 40.8 arc min against 13.3 arc min. When the two-part stimulus was vertically rotated, the triangles occupied the lower (Figure 1(e)) or upper (Figure 1(f)) positions. The strength of the overestimated height rather than the length (width) was measured. The triangles in the lower position (Figure 1(e)) show that the central orientation was approximately 12 times stronger (20.4 arc min) than the peripheral orientation (1.6 arc min). The triangles in the upper position (Figure 1(f)) exhibit a nine-fold predominance of the central orientation over the peripheral one (22.4 arc min against 2.4 arc min).

The pronounced difference between the two orientations could be attributed to the difference in the strength of the contraction effects. The contraction of the length of an angle (inward-pointing Müller–Lyer wings) is supposed to be caused by the centroid mechanism (Bulatov, 2017): the perceived position of the angle apex shifts toward the centroid. The Gaussian profile of the attention fields (Bum et al., 2023) results in a larger displacement of the vertex of the angle when it is further from the midpoint of the two-part stimulus, and consequently, in a more substantial balance of expansion of the length of the reference object. Therefore, the peripheral orientations of the angles and triangles exhibited lower errors when the length was overestimated in the experiments.

Expansion, rather than contraction, was the primary objective of this study. However, contractions are closely related to dominant targets. Angles or the inwards pointing Müller–Lyer wings that cause contraction of the perceived distance are a common contour component of various objects. Therefore, both the contraction itself and its interaction with the expansion, as active links in the physiological system, also fall within the scope of this study.

The obtained results are divided into six sections according to the spatial properties of the stimuli and the type of contour. The data are presented in the following order: Experiment I (shapes), Experiment II (luminance boundaries), Experiment III (texture changes), Experiment IV (colour contrast), Experiment V (perceptual grouping), and Experiment VI (illusory borders). Descriptions of the stimuli, results, and discussion are presented sequentially for each experiment.

## Experiment I: Shapes

The pilot observations aimed to demonstrate that stimuli formed by line contours cause an increase in relative size. The angles and triangles are similar in terms of their spatial structure and shape. However, the expansion strength varied when the contour configuration changed. The dependence of the expansion strength on stimulus shape was further tested in Experiment I using various geometric figures drawn in lines. Filled figures with no lines on the edges were used for comparison. Stimuli of limited heights and varying appearances were included.

### Stimuli

**Figure 3.**
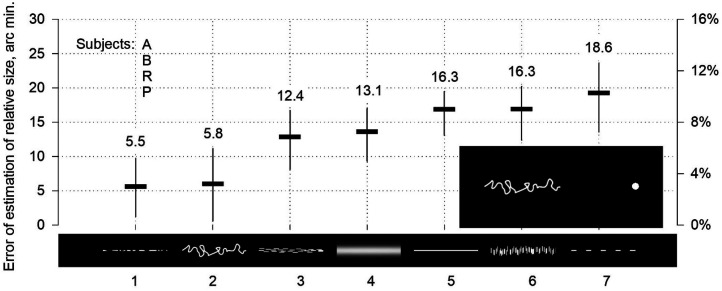
Estimation of relative length of low stimuli.

*Note.* Horizontal bars and figures above, average of experimental data (arc min) of four subjects. Vertical bars, confidence intervals. In the lower-right corner, facsimile of the entire two-part stimulus with a tangled line as reference. Facsimiles of the referential stimuli, below.

The left side of the two-part horizontal stimulus was used as the referential object presentation (Figure 2). Four geometric figures were selected as references: rhombuses, triangles with peripheral orientation, ellipses, and rectangles. The lengths of the figures are the same, at 192 arc min. The area remained the same, at 6,912 arc min². The heights of the stimuli differed: 72 arc minutes for rhombuses, 164 arc minutes for triangles, 46 arc minutes for ellipses, and 36 arc minutes for rectangles. The testing distance was limited to the sliding spot and the edge of a rectangle or triangle, an elliptical endpoint, or a rhombus vertex. Stimuli with a luminance of 76 cd/m² were presented against a background of 23 cd/m². Stimuli resembling narrow ribbons, 180 arc min long and 2.4–9 arc min high, were used (Figure 3). The stimulus and background luminance were 23 and 0.01 cd/m², respectively. The subjects adjusted the test distance to the reference stimulus length in the experiments.

### Results

According to the averaged data, an increase in length was observed without exception (Figure 2). However, the magnitudes of the errors differed. Rectangles turned out to be the most potent inducers of overestimation (21 arc min, 11% of the stimulus length); ellipses were weaker (14–15 arc min, 7.5%); triangles were even weaker (8–9 arc min, 4%); and rhombuses were the lowest (5–8 arc min, 3%). The Kruskal–Wallis test revealed a significant difference in the mean [*H*(7) = 99.242, *p* < .001]. Post hoc pairwise multiple comparison analysis (Tukey's test) was used to compare each pair of reference (Table 1). The differences between the empty and filled figures were not significant. It can be assumed that the error value depends on the shape, although there may be cases where the values are similar or nearly identical for different shapes. The errors did not rely on area or perimeter. The areas of all shapes were the same. Perimeters differed: triangles had the most extended perimeters (463 arc min), followed by rectangles (456 arc min), rhombuses (414 arc min), and ellipses (411 arc min). However, the errors for the triangles are neither the highest nor the lowest.

**Table 1.** Comparison of estimations of the relative size of different stimuli.

Stimuli	Different of ranks	*q*	*p*	*p* < .05
Contour rhombus vs. contour triangle	1226,5	0,508	1,000	No
Contour rhombus vs. contour ellipse	9354,5	3,873	0,111	No
Contour rhombus vs. contour rectangle	20323,0	8,413	<0,001	Yes
Contour triangle vs. contour ellipse	8128,0	3,365	0,251	No
Contour triangle vs. contour rectangle	9096,5	7,906	<0,001	Yes
Contour ellipse vs. contour rectangle	10,968,5	4,541	0,029	Yes
Filled rhombus vs. filled triangle	5160,0	2,136	0,803	No
Filled rhombus vs. filled ellipse	10,505,5	4,349	0,044	Yes
Filled rhombus vs. filled rectangle	23,818,5	9,860	<0,001	Yes
Filled triangle vs. filled ellipse	5345,5	2,213	0,772	No
Filled triangle vs. filled rectangle	18,658,5	7,724	<0,001	Yes
Filled ellipse vs. filled rectangle	13,313,0	5,511	0,002	Yes
Contour rhombus vs. filled rhombus	2824,0	1,169	0,992	No
Contour triangle vs. filled triangle	1109,5	0,459	1,000	No
Contour ellipse vs. filled ellipse	1673,0	0,693	1,000	No
Contour rectangle vs. filled rectangle	671,5	0,278	1,000	No

*Note.* Tukey's test.

Individually, the magnitude of the perception errors varied among subjects. In some cases, the error sign became negative: for rhombuses (subjects A, I, and G), triangles, and ellipses (subjects I and G) in Figure 2(a), and for rhombuses (I, A, G, and B), triangles, and ellipses (I) in Figure 2(b). It should be noted that contraction never exceeded expansion when empty spaces were compared to rectangles. The rectangles were always longer.

The spatial effectiveness of overestimating stimulus length, like estimation strength, depended on the shape (Figure 2). According to the averaged data, the range of variation was 2‒10%. The effectiveness was highest for rectangles (10.5%), lower for ellipses (8.5%), even lower for triangles (4.5%), and lowest for rhombuses (2.5–4.5%). The difference in the spatial effectiveness between empty and filled figures was not significant.

The low stimuli showed varying degrees of overestimation of length (Figure 3). Stimulus 7, regular strokes, was the most inducing pattern (19 arc min; 10%) and, according to Oppel's findings from 1854 to 1855 (Wade et al., 2017), slightly exceeded stimulus 5, the undivided line (16 arc min; 9%). Stimuli 7 and 5, as well as stimulus 6, rain (16 arc min; 9%), may be conditionally grouped as the strongest. Stimulus 1, irregular strokes, and stimulus 2, a tangled line, can form a group of weak patterns (5.5–6 arc min; 3%). Stimulus 3, a lake, and stimulus 4, a defocused line, were assigned to the intermediate group (12–13 arc min; 8%). These artificially formed groups illustrate the dependence of the magnitude of overestimation on the appearance of the object, irrespective of whether the magnitudes were similar or equal.

Spatial effectiveness was also related to the appearance of the stimuli and varied from approximately 3%‒10% (Figure 2).

### Discussion

**Figure 4.**
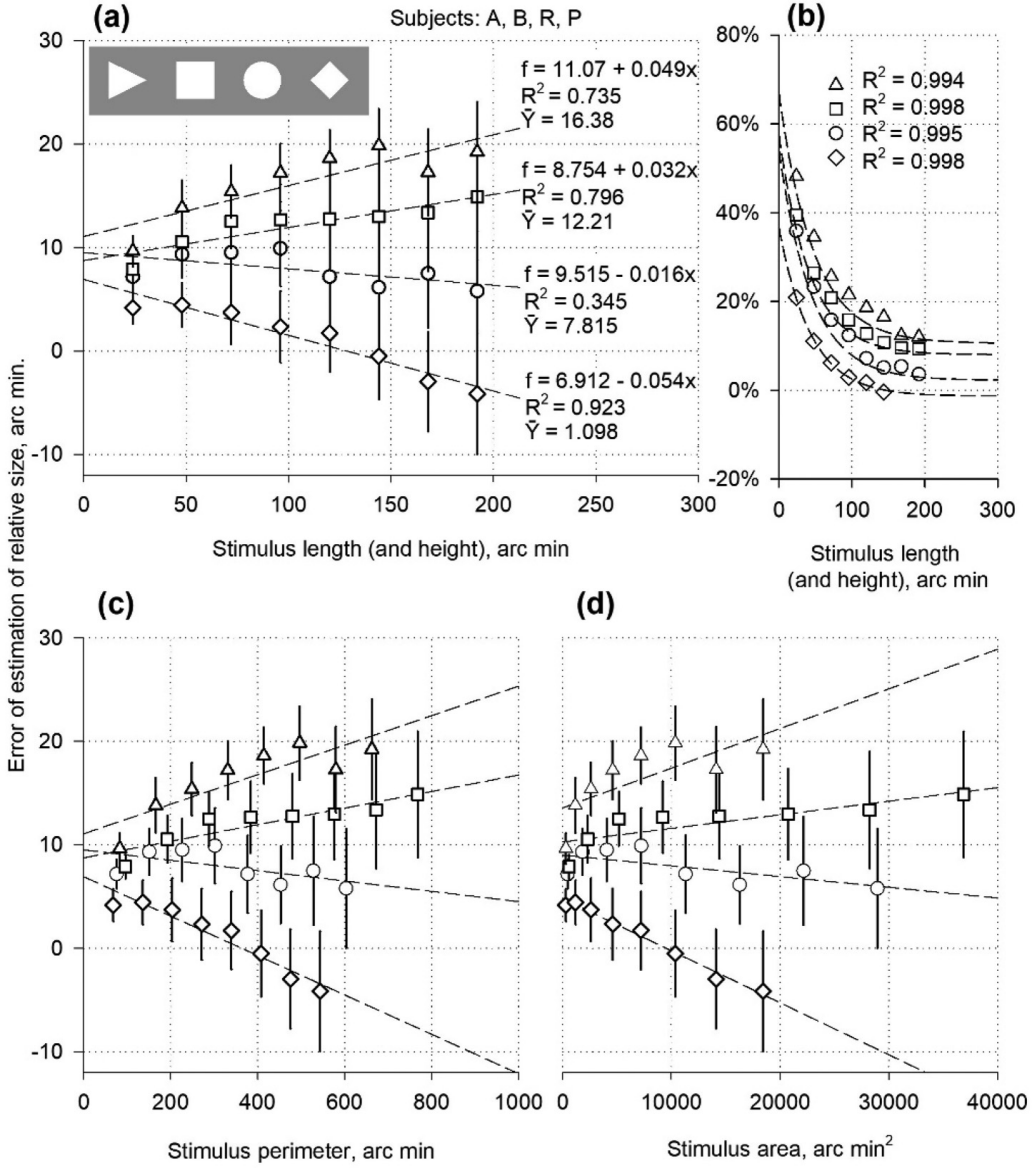
Relative size for varying length, height, perimeter, and area of luminance-contrast stimuli.

*Note.* Experimental data are shown by symbols corresponding to the stimuli in form (see facsimile images in the upper-left corner). Vertical bars, confidence intervals. Dashes in (a), (c), and (d) are regression lines, and in (b), the exponential regression.

The rectangles were always perceived as being longer or wider than their physical lengths (Figure 2 and 4(a)). No visible signs of contraction in length were observed in the experimental curves for rectangles. However, the procedure of contraction indeed contributed to the formation of the final sensory response. First, angles are the principal structural components of the rectangular shape. The pairs of angles at the ends of the diagonals should be considered as tilted Müller–Lyer wings directed inward, with centroids not situated on the horizontal stimulus axis. However, the lengths in the experiments were aligned along the horizon. Therefore, tilting could evoke cosine modulation of the Müller–Lyer contraction magnitude, as demonstrated by Brentano figures comprising the Müller–Lyer angles or arcs of a circle (Bulatov et al., 2011), resulting in a low contraction effect. Second, other observations supported the idea that contraction participates in the perception of rectangles: the errors of overestimation of the length of the rectangle varied between subjects in a range from 1% to 23% (Figure 2), which is about half of the range of the Oppel–Kundt illusion magnitude variations between 2% and 50% (Bertulis Čerkelis et al., 2018). It can be acknowledged that the Oppel-Kundt illusion is an exclusive example of the true expansion of distance since there are no angles, arcs, or other visible causes of the contraction effect in the stimulus structure. In comparison, the illusion of the increased rectangle length is not exclusive to expansion. A low contraction occurs due to the wings effect when assessing the length of the rectangles. The summation of two competing processes, originating from different neural mechanisms, emphasises the more vital member, expansion, and results in the final perceptual event: an overestimation. The contraction–expansion ratio is low (C/E < 1).

In the triangles and rhombuses, the vertices were oriented horizontally along the stimulus axis. The vertex components in the contour could affect the perceived length under full force, as the inward Müller–Lyer wings would noticeably reduce the magnitude of the expansion. The contraction effect in rhombuses was more substantial than that in triangles, likely due to the two pairs of angles (wings). One pair of wings in the triangles may have a more significant impact than the two arcs of high curvature at the ends of the ellipses. However, the contraction is more pronounced in the ellipses than in the rectangles.

According to the data, contours may be the primary factor contributing to size estimation distortions. Contours of any shape used in Experiment I, as in the pilot experiments, could cause two opposite types of perceptual distortions that balanced each other during the length-matching task. The strength of misperception of both types and summation coefficients varied between subjects, as in the summation of two expansion effects caused by the outward wings of the Müller–Lyer figure and the filled part of the Oppel–Kundt stimulus (Bertulis Čerkelis et al., 2018). For example, four subjects judged the filled rhombus to appear shorter than it was (Figure 2). The reason for this could be a relatively strong contraction (C/E > 1), which led to a reduced perceived size. Conversely, the other six subjects perceived the rhombus as longer. The dominance of expansion over contraction (low contraction/expansion ratio; C/E < 1) could lead to an increased perceived size. These data demonstrated contraction–expansion interrelations in dependence on individual properties of visual processing. The data for the filled rectangle looked different. All 10 subjects, without exception, perceived the stimulus as longer. The shape of the stimulus altered the result. To evaluate the impact of the shape more fully, one should consider not individual responses, but averages. The mean values arrange four different shapes into a row of increasing strength of overestimations: diamond (4.6–7.8 arc min), triangle (8.2–8.5 arc min), oval (13.5–14.7 arc min), and rectangle (21.2–21.3 arc min), both filled and empty outlines. The data suggest that the outline forming the shape is the primary factor determining the laws of contraction–expansion interaction. Individual properties contribute to a certain extent.

The type of object edge did not affect perception distortions. The difference in distance overestimation values between the empty and filled figures was not significant (Table 1). Similarly, the differences in spatial effectiveness were also not significant. The homogeneous surface of objects seemed to have a weak influence, if any, on the overestimated value.

Ribbon-like stimuli (Figure 3) had an elongated shape with a limited height; however, differences in appearance were easily seen. The stimuli demonstrated an expansion. In perception research, low stimuli, including lines, can be classified into a group of elongated rectangular shapes (Kogo & Wagemans, 2013). However, no angles at the ends of the stimuli in Figure 3 could be discerned; therefore, the Müller–Lyer contraction could not arise. Thus, all seven low stimuli, by analogy with the Oppel–Kundt figure, allowed for the measurement of the true strength of expansion. The lengths of the stimuli were the same, but expansion strengths differed. The shape and spatial structure determined the magnitude of expansion.

## Experiment II: Edges of Luminance

Edges define the spatial relationships of places where surface properties such as luminance, colour, or texture suddenly change. Edges and contour lines create a sense of integrity in the outlined area, synthesise the idea of the object's presence, and play a leading role in the perception of form. The luminance edges and contour lines are equally blamed for the expansion of relative size. The data from Experiment I demonstrated no significant differences between the manifestations of expansion for the line contours and the luminance borders of the rectangles, triangles, and ellipses. To confirm these results, luminance-contrast stimuli of various sizes (length, height, perimeter, and area) were tested.

### Stimuli

At a luminance of 76 cd/m², the reference objects – filled squares, circles, rhombuses with hypotenuses of equal length (i.e., squares tilted by 90°), and isosceles triangles with the central orientation – were visible against a background of 23 cd/m² luminance. The height and width of the reference objects were the same and varied simultaneously from 24 to 192 arc min during the presentations. The perimeter and area changed within the 68–768 and 288–36864 arc min^2^ ranges, respectively. The test distance was limited to a sliding spot for stimuli consisting of discs, rhombuses, and triangles or a sliding vertical stripe for stimuli with squares. The heights of the stripes and squares varied in parallel. The participants matched the test distance with the width of the reference object.

### Results

Size-matching errors were observed for all stimuli and participants (Figure 4(a)). The minor stimuli (24 × 24 arc min) caused a 4–9 arc min overestimation that increased monotonically, reaching 15 arc min and 20 arc min with the increase in the size of squares and triangles, respectively. The contraction/expansion ratio gradually decreased. In contrast, the error value decreased with the enlargement of discs and rhombuses, and even became negative for large rhombuses (163 × 163 arc min and 192 × 192 arc min). The contraction/expansion ratio increased. It reached a value of 1 (the error decreased to zero) for rhombuses with a size of approximately 140 × 140 arc min. In general, the contraction/expansion ratio largely depended on the shape: rhombuses had the highest ratio, discs had somewhat lower, squares even lower, and the triangles of the central orientation showed the lowest.

The spatial effectiveness of misestimating decreased with increasing stimulus size (Figure 4(b)). All the curves showed an exponential profile. However, the curves of the triangles and squares in Figure 4(b) reflect the expansion process to a greater extent. In comparison, the curves of the discs and rhombuses reflected the contraction process to a greater extent.

### Discussion

In the present experiments, the luminance edges formed the shapes of the reference stimuli and determined the signs and strengths of the physiological distortions of perceived relative size. As observed in Experiment I, the perimeter and area of the figures did not affect the expansion strength. For any fixed perimeter or area, the continuity of the increasing values of misestimating did not vary: rhombuses, discs, squares, and triangles with the apex oriented centrally (Figure 4(c) and (d)). The narrow integration of excitations along the contour circumference is neither a sufficient nor obligatory condition for discriminating shape and relative size. In a contour-based analysis of shape (Kimia et al., 1995), the contour can be expressed as a parameterised distance function along the contour path rather than a perimeter function. Human sensitivity is low for area estimation compared to the high sensitivity for aspect ratio (Morgan, 2005) and relative size.

The effectiveness of the overestimation was approximately 20% for a reference rhombus measuring 24 × 24 arc min, but decreased to 0% for larger stimuli (140 × 140 arc min). Within the same size limits, the effectiveness of the triangle decreased from 50% to 17%. The dynamics of overestimation effectiveness corresponded, to some extent, to the foveal and parafoveal regions of the retina. The mechanisms involved in relative size processing may be related to the cortical areas of central vision.

The results obtained illustrate variations in the contraction/expansion ratio as the size of the referential stimulus changes. Expansion occurred when testing any stimulus but was more pronounced with squares and triangles. Larger sizes yielded higher errors (Figure 4(a)). Contraction could also be contributing to the mistakes in testing all figures but it was more evident in the data for discs and rhombuses. Larger sizes yielded lower errors (Figure 4(a)). The discs and rhombuses may have produced a relatively strong contraction effect due to the presence of pairs of angles or arcs functioning as the inward-facing Müller–Lyer wings. The contraction was stronger for rhombuses than for discs, presumably because of the straight lines of the wings. The triangles in the central orientation have one pair of wings. They produced a relatively weak contraction, as indicated by the data from the pilot experiments (Figure 1(b)). Two pairs of wings in rectangles produced cosine-modulated contraction (Experiment I), which appeared more potent than that in the triangles with the central orientation.

Rectangular stimuli were used in further experiments because they created a relatively low contraction/expansion ratio compared to other geometric shapes possessing symmetry (Experiment I).

## Experiment III: Texture-Induced Contours

Stimuli with random geometric figures appear larger than those with uniform areas (Verrillo & Graeff, 1970). Textured rectangular patterns, compared to uniform greyscale areas and distances within objects versus background regions, exhibited overestimations (Giora & Gori, 2010; Vickery & Chun, 2010). In Experiment III, texture-defined borders were used to investigate whether this contour type created an expansion effect, similar to the experiments with line contours and luminance contrasts. Texture edges, like line contours, synthesise the idea of an object's presence, shape, and integrity.

### Stimuli

**Figure 5.**
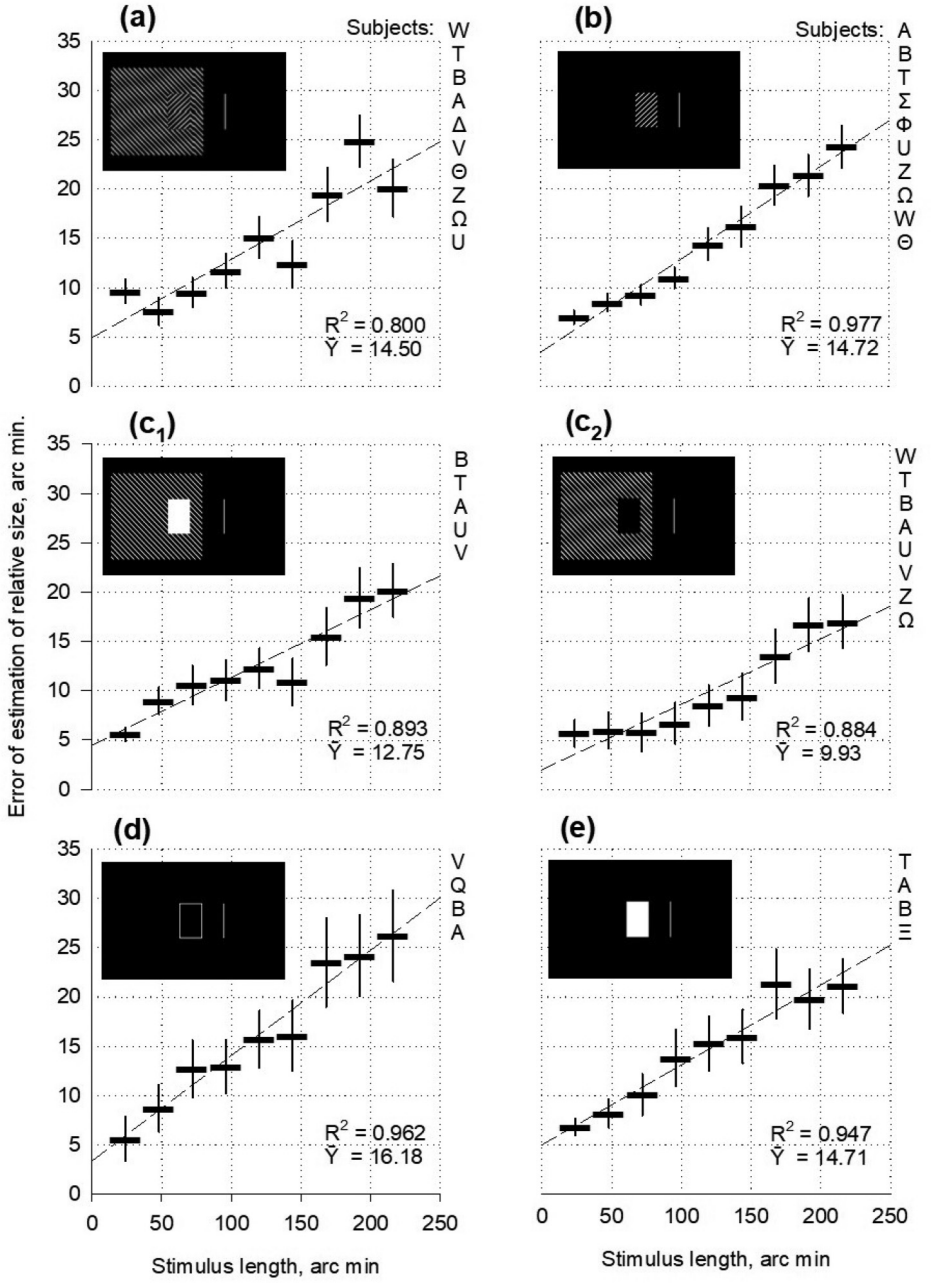
Relative size as a function of the length of texture-contrast and luminance-contrast stimuli.

*Note.* Facsimile images of the stimuli are shown on the corresponding graphs. Horizontal bars, the average of experimental data. Vertical bars, confidence intervals. Dashes, regression lines. $\bar{Y}$ , mean value, arc min.

Synthetic textures of diagonal lines with a spatial frequency of six cycles/degree were used to create referential stimuli (Figure 5): (a) a rectangle and a background that differed from each other in the orientation of the lines, (b) an isolated texture stimulus, and dark (c_1_) or light (c_2_) rectangular windows framed by the texture. Contoured and filled rectangles are used for the control (d) and (e). The rectangle (a) was defined by the boundaries of texture signals; rectangles (b), (c_1_), and (c_2_) were formed using a combination of texture and luminance features, and rectangles (d) and (e) were created using luminance borders. The test distance was limited to the sliding vertical bar and background texture edge in (a), (c_1_), and (c_2_), or the referential rectangle border in (b), (d), and (e). In the experiments, the lengths of the reference rectangles varied from 24 to 216 arc min. The height was fixed at 120 arc min. The background luminance was 0.01 cd/m^2^, and the luminance of the stimuli was 0.38 cd/m^2^. The participants evaluated the perceived length along the horizontal axis of the rectangles by performing a length-matching task.

### Results and Discussion

Overestimation of the size was observed in all cases (Figure 5). In all subjects, the stimuli indicated a pronounced predominance of expansion over contraction. The regression lines in Figure 5 illustrate the growth of overestimation as the length of the objects outlined by changes in texture and luminance increases. The error value increased similarly for the borders of all specified types: texture (a), texture and luminance (b, c_1_, and c_2_), line contours (d), and luminance contrast (e). The differences between the mean values of 14.5 arc min (a), 14.7 arc min (b), 12.8 arc min (c_1_), 9.9 arc min (c_2_), 16.2 arc min (d), and 14.7 arc min (e) were considered statistically insignificant according to the Kruskal–Wallis H test, *H*_(5)_ = 17.068, *p* = .004. Post hoc tests (Dunn's method) revealed a dissimilarity between the contour rectangle (d) and the empty window framed by the texture (c_2_), *p* = .063. No other pairs showed significant differences. More data must be collected to determine whether the edges of the texture framing an empty rectangle (c_2_) caused less expansion than the contour lines of the same unfilled stimulus (d). However, the existing results are sufficient to suggest that the contrast contours of the texture lead to a visually noticeable increase in length.

## Experiment IV: Colour Contrast Outlines

Geometric optical illusions have often been presented in achromatic (black and white) form; however, chromatic variants have been tested (Cavanagh, 1991). Some findings (Livingstone & Hubel, 1987; Surkys, 2007) claim that geometric optical illusions vanish under conditions of isoluminance (no luminance difference between the stimulus and its background, only difference in hue), but others (Hamburger et al., 2017) have shown that illusions of length (Müller–Lyer, Ponzo, and vertical/horizontal), size (Delboeuf, Ebbinghaus), and orientation (Hering) persist under such isoluminant conditions. To investigate the chromatic factors contributing to the effect of increased relative size, coloured objects were tested in Experiment IV with the length adjustment procedures.

### Stimuli

The reference objects (red and blue rectangles) were displayed against a green background with a luminance of 2 cd/m² on an OLED48C11LB monitor. The background colour was obtained using a green emitter. The colours of the stimuli were a mixture of two parts, each in equal amounts, from the red, green, and blue emitters. Equality between colours in luminance was established using the technique of minimal movement (Anstis et al., 1983) for each subject. Perceptually, the rectangles were distinguished only by a difference in hue from the surroundings, whereas the luminance levels were equal. The heights of the reference rectangles and terminal stripes were 120 arc min. The colour of the terminal stripe matched that of the presented rectangle. The rectangular length in the presentations varied from 24 to 216 arc min.

### Results

**Figure 6.**
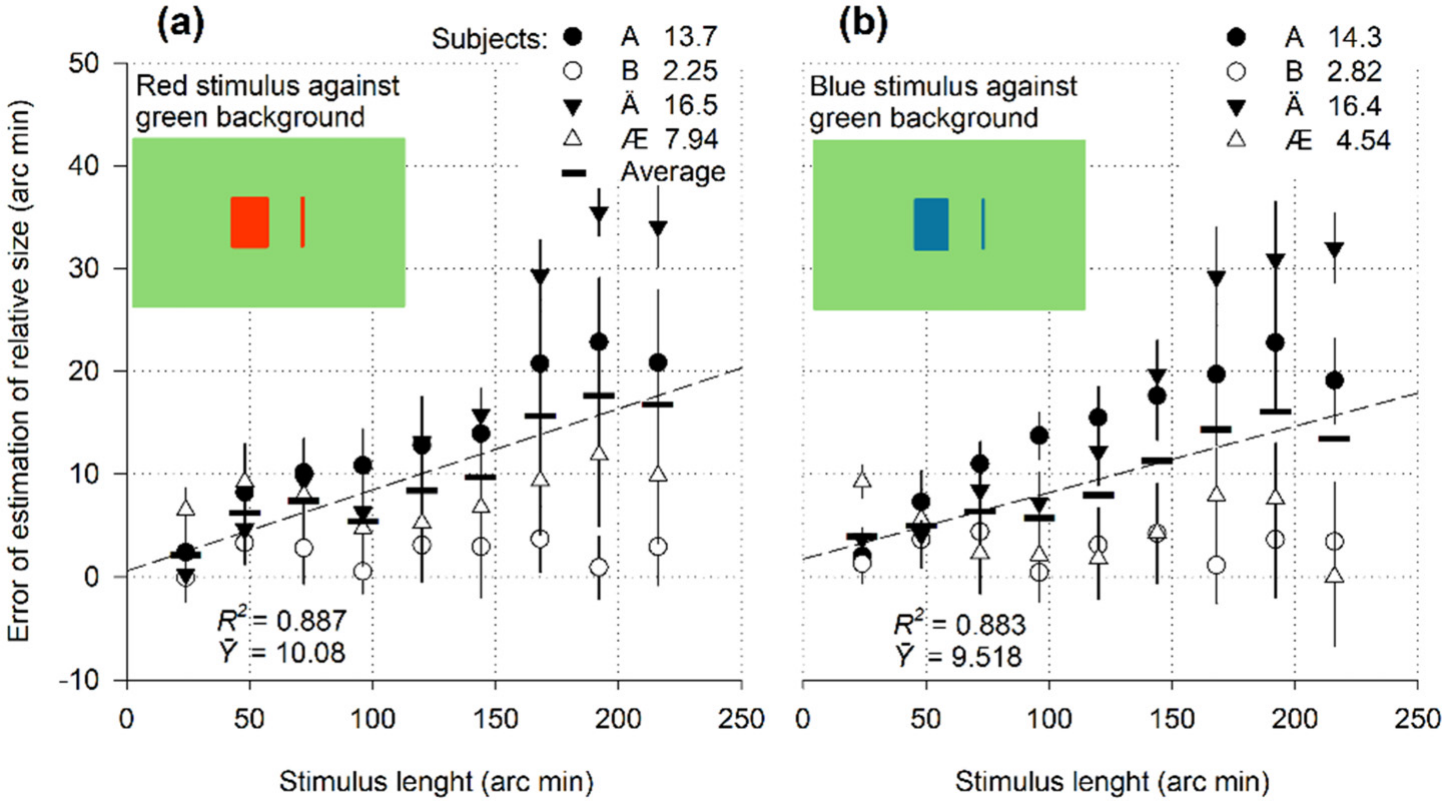
Relative size as a function of the length of colour stimuli.

*Note.* Facsimile images of the stimuli are shown on the corresponding graphs. Horizontal bars are an average of experimental data. Symbols, individual data of subjects. Dashes, regression lines. Vertical bars, confidence intervals. $\bar{Y}$ , mean value, arc min. Figures in the upper right corner show individual averages for each subject.

**Figure 7.**
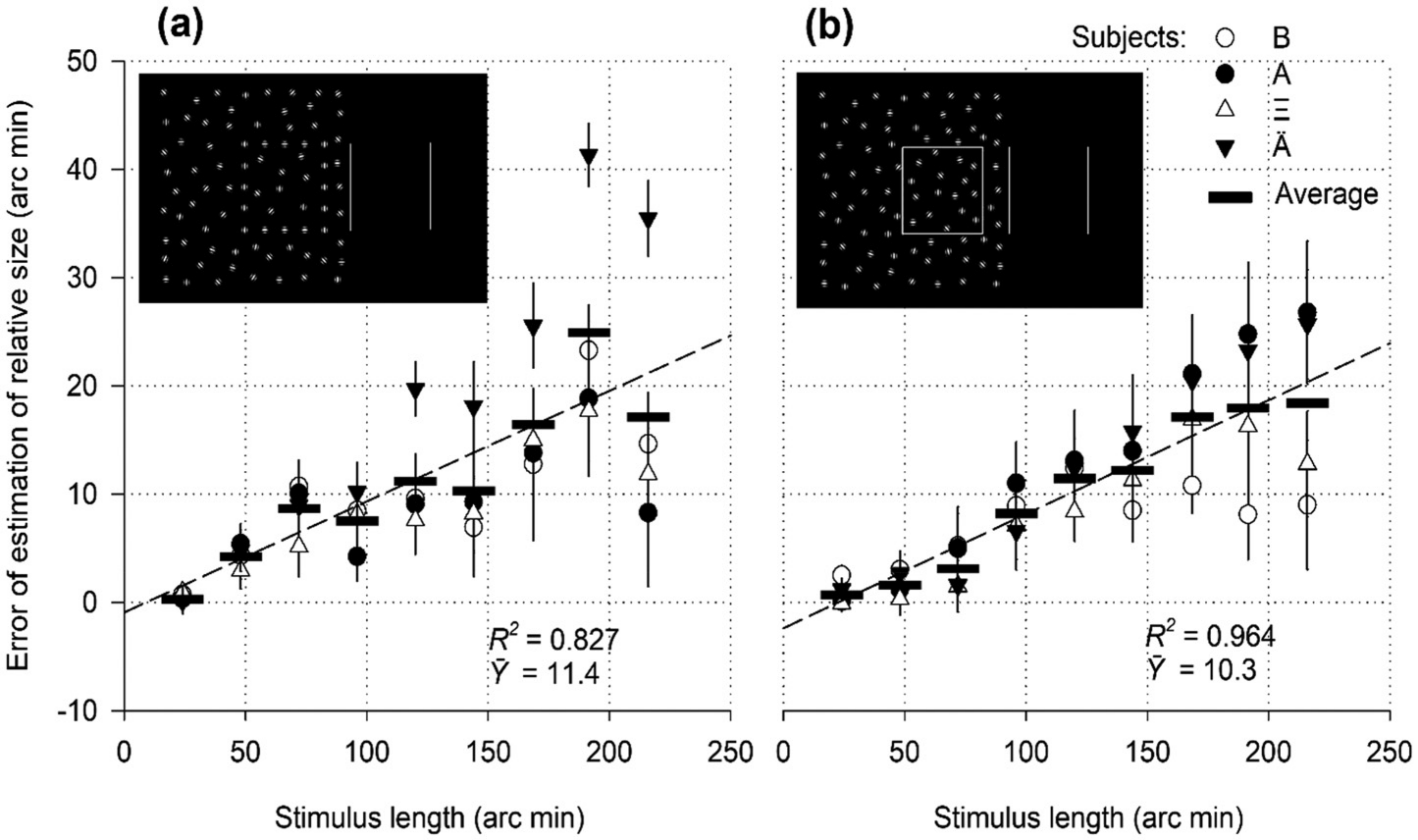
Relative size as a function of the length of contour formed by aligned elements.

*Note.* The stimulus of perceptual grouping is shown in a facsimile image (a), and the control stimulus is shown in (b). Horizontal bars, average of experimental data. Symbols, individual data of subjects. Vertical bars, confidence intervals. Dashes, regression lines. $\bar{Y}$ , mean values, arc min.

Observers could see the stimuli, their shapes, individual angles, and sides. The effect of size expansion occurred for both the red and blue rectangles and was approximately equal in strength (Figure 6). The overestimation errors gradually increased with increasing lengths of both red/green and blue/green stimuli, as was the case with white/black stimuli (Figures 2 and 3). The mean values $\bar{Y}$ for the colour rectangles (10.08 arc min and 9.518 arc min) did not differ significantly; Wilcoxon signed-rank test: *Z*(378) = 0.382, *p* = .703. The strength of the illusion varied considerably among different subjects. For instance, the average values for subject B in Figure 6 were low, 2.25 arc min (red rectangle) and 2.82 arc min (blue rectangle), but for subject Ӓ, they were high, 16.5 and 16.4 arc min. Therefore, the difference between distortions in chromatic and achromatic rectangles [9.52 arc min in Figure 6(b) vs. 14.71 arc min in Figure 7(е); Student's *t*-test: *t*(16) = –2.197, *p* = .043] may not be considered physiologically meaningful.

### Discussion

The data demonstrated the expansion of visual objects under conditions of absent luminance contrast. The stimuli were visible solely due to the colour contrast. Based on the resulting experimental curves, colour contrast affects the estimation of an object's relative size in the same way as the contrast of texture and luminance. Wavelength-sensitive pathways start from their receptive fields in V_1_ and V_2_ and run parallel to achromatic pathways. It can be assumed that the size distortions under consideration rise above the early levels of the cortical system. The joint processing of chromatic and luminance-defined contours occurs well above the level of early visual areas (Hamburger et al., 2017).

## Experiment V: Contours of Perceptual Grouping

Dots, spots, or dashes aligned at a certain distance from each other, but embedded in the noise elements of random positions, can create a stable impression of a line or a straight edge. Information sampled from different cortical locations is integrated to obtain the perception of an extended object (Barlow & Reeves, 1979; Beck et al., 1989; Field et al., 1993; Smits et al., 1985). Visual mechanisms can efficiently summarise information from signal elements throughout space (Loffler, 2008). However, do contours synthesised using grouping procedures lead to relative size distortions?

### Stimuli

Gabor patches (a sinusoid enveloped by a Gaussian window), visible as rounded shapes with a diameter of approximately eight arc min, were used as signalling elements for the formation of the imaginary edges of the referential stimuli (Figure 7(a)). The elements were located along the perimeter of a rectangle and oriented according to its vertical and horizontal sides. The corner patches had vertical slopes. The distance between the centres of the vertically aligned elements was 30 arc min. The distance between the horizontally oriented elements varied from 16 to 32 arc min, depending on the increase in the length of the imaginary edge. Gabor patches of random orientation were distributed randomly in the background with a density of 2.8 elements per degree squared. The subjects easily identified the rectangular shape of the stimulus (Figure 7(a)) when presented on the monitor screen for monocular observation.

Rectangles formed by the line contours were used as controls (Figure 7(b)). The height of all referential rectangles was 120 arc min, and the length varied from 24 to 216 arc min in the presentations. The test part was limited to two vertical stripes with a height of 120 arc min and was located on the right side of the reference.

### Results

All participants experienced the illusion of an enlarged size with a magnitude of 3–5 arc min when exposed to a stimulus 48 arc min in length (Figure 7(a)). When the stimulus length was extended to 192 arc min, the illusion gained strength and reached a value of approximately 20 arc min. The mean value of the illusion, $\bar{Y}$ , was 11.4 arc min. For subject Ӓ, the illusion grew faster than for other subjects. The manifestation of the illusion caused by the control stimulus (b) was nearly identical to that of the stimulus (a): the maximum value approached approximately 19 arc min, and $\bar{Y}$ was 10.3 arc min. The two datasets were closely related: *U*_(432,756)_ = 156,828.0, *p* = .255, and to the other sets presented in Figures 5(a) and 6(a): *U*_(396,756)_ =  112,468.0, *p* < .001 and *U*_(360,756)_ =  124,506.0, *p* = .021, respectively.

### Discussion

Perceptual grouping resulted in a rectangular shape, which led to distortions in the perception of relative size. The nature of the overestimations was similar to that determined by the same shape, a rectangle, but was represented in other neural structures than those sensitive to wavelengths, coded for luminance contrast, and tuned to changes in texture. This was consistent with the assumption that distance comparison and errors in overestimating the relative size occur above early areas in the cortex.

## Experiment VI: Illusory Contours

Discontinuous borders, such as incomplete contour lines, can be perceived as objects that are partially covered by others. Missing borders can be supplemented by surrounding unclosed fragments arranged in an approximately collinear or aligned order (Grossberg, 2017). A whole can be created by merging real and illusory borders (Kanizsa, 1955; Roncato, 2012). Illusory (subjective) contours are more informative than luminance contours, as subjective contours are always perceived as the boundaries of a closer surface (Gillam, 2017) and help determine the depth and completeness of forms in a 3D landscape. The question is whether subjective (non-existent) segments create the illusion of increased size, such as the contours of lines, aligned signal elements, and borders of texture, luminance, and colour.

### Stimuli

**Figure 8.**
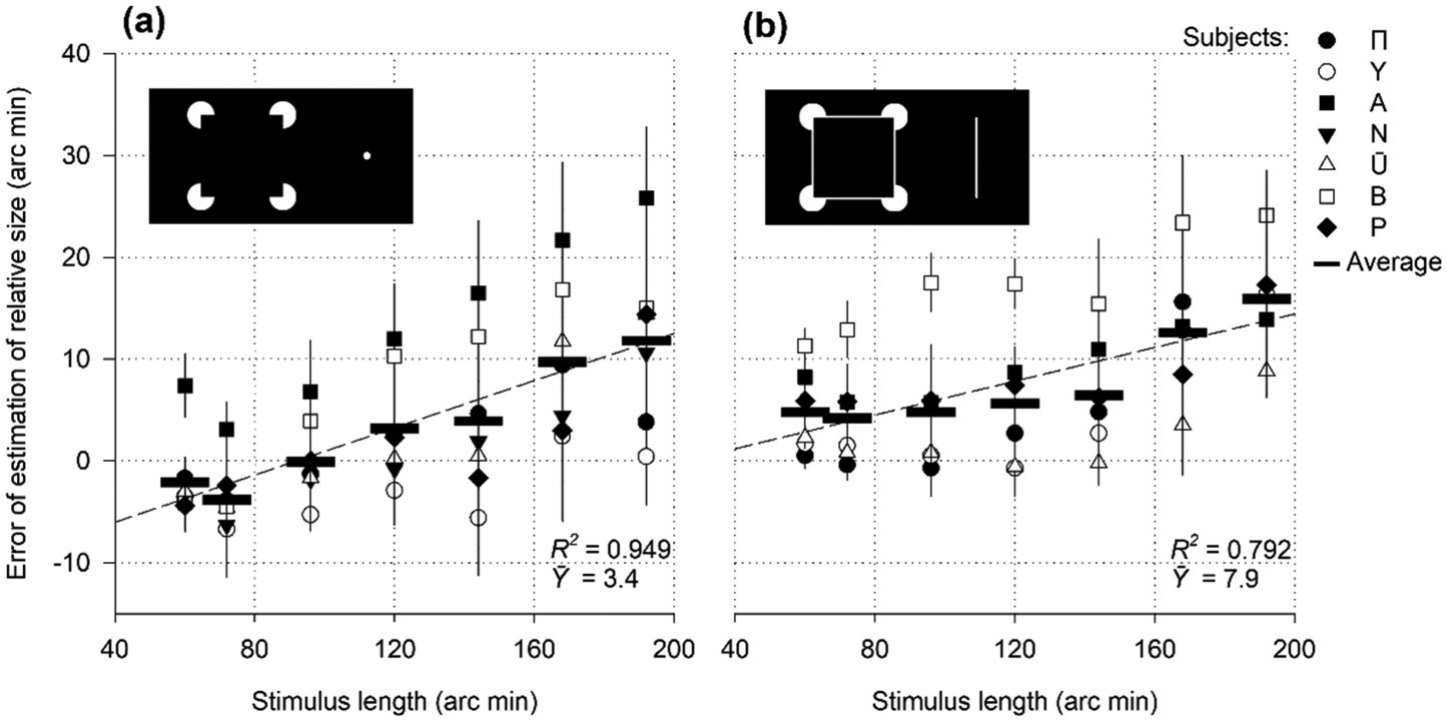
Relative size as a function of the length of the stimulus of the illusory contours.

*Note.* Kanizsa-type stimulus is shown in a facsimile image (a), and the control is shown in (b). Horizontal bars averaged experimental data. Symbols, individual data of subjects. Vertical bars, confidence intervals. Dashes, regression lines. $\bar{Y}$ , mean values, arc min.

**Figure 9.**
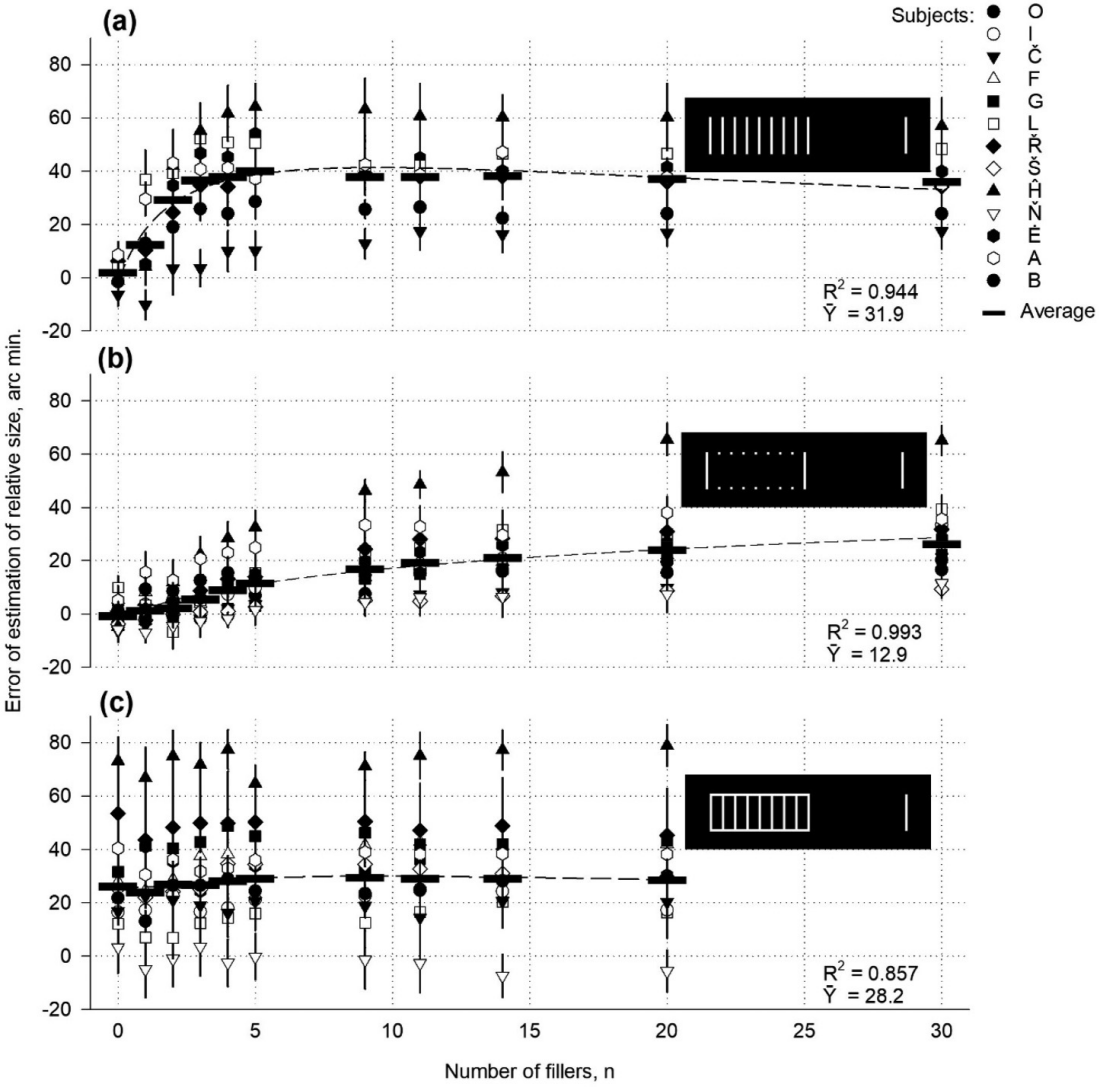
Relative size as a function of the number of filling elements in the referential part of the divided stimulus.

*Note*. The usual Oppel–Kundt stimulus is shown in a facsimile image (a), a modification devoid of filling stripes but having endpoints is shown in (b), and a structure with two additional horizontal lines in (c). Horizontal bars, average experimental data. Symbols, individual data of subjects. Vertical bars, confidence intervals. Dashes, the log-normal regression in (a) and (c), and the exponential regression in (b). $\bar{Y}$ , mean value, arc min.

The Kanizsa rectangles (Figure 8(a)) and Oppel–Kundt figures (Figure 9(a)) were tested experimentally. Contour rectangles were inserted into the Kanizsa pattern as a control (Figure 8(b)).

The height of the Kanizsa rectangles was 144 arc min, and the length (width) varied from 60 to 192 arc min. The radii and luminance of the bright discs were 24 and 76 cd/m^2^, respectively. The luminance of the lines and spots was 76 cd/m^2^. The background luminance was 23 cd/m^2^.

The filled part of the Oppel–Kundt stimulus (Figure 9(a)) measured 72 × 192 arc min. The number of filling stripes varied from 0 to 30. The stripe luminance was 76 cd/m^2^. The background luminance was 23 cd/m^2^. In the modified Oppel–Kundt stimuli, the filling stripes were removed, but their endpoints remained (Figure 9(b)), or two horizontal contour lines were added (Figure 9(c)).

### Results

Curves of the upward slope were obtained (Figure 8). For observer A, the magnitude of overestimation gradually increased from 3 to 26 arc min as the stimulus length increased from 60 to 192 arc min. The other six observers reported smaller perceived lengths (widths) of the narrow rectangles (60 and 72 arc min). Errors even have a negative sign. However, the underestimation weakened and approached zero when the rectangles widened. Moreover, the errors became positive for wider rectangles and continued to increase with further elongation. Taken together, the experimental points formed an ascending queue for all subjects. The upward slope was similar to that obtained in previous experiments using rectangles (Figures 1, 4 to 7).

With the help of the control stimulus (Figure 8(b)), all subjects presented positive error values in the ascending profile. Observer A perceived the distance between the subjective boundaries in the Kanizsa Rectangle to be greater than that in the drawings of the stimuli (Figure 8(a)).

In experiments with the Oppel–Kundt stimulus according to the classics, the perceived length of the divided interval first increased from zero to a maximum with an increasing number of stripes (Figure 9(a)). It then decreased slightly, corresponding to a lognormal distribution (*R*^2^ = .946). If the filling stripes were removed from the stimulus, but their endpoints remained, the perceptual errors did not disappear, and only the experimental curve decreased. The mean value lowered from 31.9 to 12.9 arc min, and the maximum was almost smoothed (Figure 9(b)). The data show an exponential increase (*R*^2^ = .993). Perceptual errors were also present when two horizontal lines were added to the filled Oppel–Kundt stimulus interval, forming a contoured rectangle. However, they remained approximately stable with various numbers of the filling stripes (0‒20). The log-normal distribution approached a horizontal line (*R*^2^ = .857; Figure 9(c)).

### Discussion

In the Kanizsa stimulus, the cut-outs of the four circles produced a sensation of a full shape, that is, a rectangle. The shape, in turn, creates a sense of distance, which can be likened to the distance to a natural light spot. The shape results directly from the interpolation, and the relative size is determined by the shape. The reverse sequence hardly occurred; the relative distance could only be imagined once it was legitimised as a feature of the surface of the imaginary rectangle.

The primary finding of the present experiments is that illusory contours induce an expansion effect, and the dynamics of interaction between expansion and contraction processes determine the perceived relative size by an observer. For instance, subject A shows an increased relative length of all Kanizsa rectangles in the experiment (Figure 8(a)). Expansion apparently exceeded contraction in all cases (C/E < 1). In the subjects’ B data for narrow rectangles, the expansion was probably relatively low, and the cosine-modulated contraction exceeded it (C/E > 1). However, for wider rectangles, the expansion effect intensified, and the C/E ratio became less than 1. In subjects P and Y, the contraction over dominance apparently manifested in a broader range of widths.

The control stimulus elicited a positive illusion sign in nearly all subjects for all stimulus sizes (Figure 8(b)). The average overestimation of line contours was twice as substantial as that of subjective boundaries (7.9 vs. 3.4 arc min). The Mann–Whitney *U*-test revealed a significant difference, *U*_(42,49)_ = 623.00, *p* = .001. The illusory and natural contours may not be physiologically identical. The neural representations and mosaics of specific cortical excitations may differ.

The decisive feature of the Oppel–Kundt stimulus was its horizontal illusory borders induced by the end spots of the filling stripes due to perceptual grouping. The subjective borders elicited the expansion of the perceived distance, like in Experiment V. The feeling of an imaginary outline became more potent, and, therefore, the illusion increased as the number of horizontally aligned spots increased (Figure 9(b)). The filling stripes are auxiliary segments that refer to the surface properties and can add to the expansion strength (Figure 9(a)). In the rectangles of line contours, the variance in the number of stripes had a negligible effect on the illusion (Figure 9(c)). The data confirm the assumption that the famous Oppel–Kundt illusion is an individual case of a general sensory phenomenon. The Oppel–Kundt expansion is sometimes called the illusion of interrupted extent (Sanford, 1903), filled space illusion (Lewis, 1912), or filled/empty illusion (Wackermann & Kastner, 2010). However, this can be acknowledged as an individual case of the perceived size expansion of an object due to contour processing.

## General Discussion

Data from six experimental series support a little-known phenomenon in visual physiology: expansion of the perceived relative size of visual objects. Within 13,110 stimulus presentations, 12,220 stimuli clearly showed an overestimation of the target distance. The participants estimated stimuli of various shapes, structures, and sizes as being longer or higher (hence, larger). However, the experimental values of overestimations did not accurately reflect the true strength of the expansion effect, but rather exposed the result of the summation of two opposing processes – expansion and contraction – originating from different neural mechanisms. In the given cases, expansion was a stronger member, thus providing a more substantial contribution to the final perceptual event. The integration coefficients of contraction and expansion can vary between different subjects and different stimuli. This is likely why the overestimation ranged from 5 to 20 arc min (Figure 2, with an average of 10 subjects); however, in a separate case, the upper limit exceeded 40 arc min.

The true magnitude of expansion could be measured in the experiments only by testing the Oppel–Kundt figure and low-ribbon-like stimuli, owing to the absence of contraction (Experiments I and VI). Contractions may not have occurred because no corners were present in the stimuli. Therefore, the Oppel–Kundt figure generated an impressive strength of the expansion effect (Experiment VI). The maximum value achieved with the stimulus was 40 arc min (average: 13 subjects). For one individual, it was 60 arc min. The spatial efficiency was 21% and 31%, respectively. The Oppel–Kundt expansion has previously been called an illusion of interrupted extent (Sanford, 1903), the filled space illusion (Lewis, 1912), or the filled/empty illusion (Wackermann & Kastner, 2010), but in the context of the present study, it ought to be acknowledged as an individual case of a general sensory phenomenon: the object's perceived size expansion.

The group of low stimuli was equal in length but differed in spatial structure, demonstrating pure expansion strength that varied from 5 to 19 arc min (average of four subjects). Spatial efficiency ranged from 3% to 10%.

A total of 890 presentations (7%) demonstrated an underestimation of length or height. The distances seemed shorter than they were. Based on the assumption of integrating opposing processes, the error values can be considered the result of the summation of contraction and expansion. This was an expansion that could be overshadowed (C/E > 1), yet it was present in all observed underestimating situations. Rare individual data on distance underestimation did not contradict the general assumption that expansion inevitably occurs when testing any object during the size-matching procedure. The mean values of the strength of the expansion ( $\bar{Y}$ ) have a positive sign in all experiments, with 38 observers optionally participating in the experiments.

The number of visual objects used in the present experiments (29, excluding size options) was negligible compared to the number of possible visual forms in the world. However, the results obtained unambiguously support the grounds for considering the effect of an object's relative size expansion as an inherent property of visual perception. The statistical power of the expansion phenomenon calculated by using $\bar{Y}$ is 0.984, with a Type I error probability of α = 0.0159 (Cohen’s *d* = 1.6), using G*Power software.

The experimental results provide insights into the structures of different objects that could serve as a common cause for their relative size expansion. Different stimuli shaped by contour lines cause an overestimation of value variance. Among the few geometric forms, rectangles provided the highest value, circles or ellipses supplied somewhat lower values, and rhombuses generated the lowest (Experiment I). The expansion strength for the triangles and angles varied depending on their orientation within the two-part stimulus (pilot test and Experiments I and II). The spatial effectiveness of the expansion varies with the strength of the expansion. Filled-in geometric figures with no lines on their borders exhibited similar expansion strengths and spatial effectiveness variances. Likely, a homogeneous filling did not result in any changes. The change in outline type from lines to a sharp luminance contrast did not influence the result of the distance assessment. Moreover, an exact shape, such as a rectangle with different contour types (characterized by abrupt changes in luminance, colour, and texture or depicted by lines, perceptual grouping, and illusory outlines), yielded similar overestimation values (Experiments I, III, IV, V, and VI). Increasing the size of rectangles with various outline types resulted in similar profiles for the overestimation curves. Differences in the structure of the lines and ribbon-like stimuli, with fixed lengths, resulted in unequal expansion strengths. A modified Oppel–Kundt figure with no stripes enabled expansion. The sequences of regular spots at the horizontal edges of the figure (endpoints of stripes) were sufficient for the illusion to manifest. The stripe endpoints help form a subjective contour. The strength of the illusion of spots was lower than that of stripes. The stripes played a role in the surface attributes, adding to the expansion, as opposed to the homogeneous surface, which did not strengthen the expansion. A certain number of stripes is more effective than a solid filling (Ebbinhaus, 1902) due to attractive/repulsive interactions between stripes (Eriksson, 1970) or terminal repulsion of the penultimate element (Mikellidou, 2012).

The data confirmed that the stimuli's subjective or factual contour was the primary inducer of expansion. The contour configuration determined the expansion value. The contour perimeter and object area did not affect the strength of the expansion. For any fixed perimeter or area, the continuity of the increasing values of the overestimation of various shapes remained the same: rhombuses, discs, squares, and triangles with the apex oriented centrally (Experiment II).

The Müller–Lyer, Brentano, and Delboeuf illusions refer to specific stimulus structures, such as pairs of inward-pointing wings, arcs, and staples, which induce the contraction of perceived distance, a process opposite to expansion. The present study confirmed the mutual influence of these two effects on the final perceptual result. Angles and arcs, as structural components of the stimulus, noticeably reduced the magnitude of expansion. Contraction may even become dominant over expansion. In contrast, presenting an angle as an independent stimulus resulted in an increase in perceptual size, with expansion prevailing over contraction. This led to the idea that functional relationships between the contour parts form the perception of shape and size rather than an integration of reactions to each part.

In general, the processing of contour excitations can result in a side effect – overestimation or underestimation of the relative size in an experiment.

Are there possible neural correlates for the overestimation of relative size in the visual system? The area of dynamics of the effectiveness of overestimation corresponded, to some extent, to the foveal and parafoveal regions of the retina. The mechanisms involved in relative size processing may be related to the cortical areas of central vision. Experimental data obtained using contours of various natures allows corresponding hypothetical reasoning on a mechanism functioning at higher levels of the visual system that could be responsible for the expansion effect. In a network of associative spatial filtering receptive fields, representations of the terminals of a stimulus (e.g., a rectangle) can be repulsed from each other because they are related to the representation of the overall contour of the referential object. The attention fields of the Gaussian profile can enhance positional shifts. Errors in the representation of positions can lead to errors in the sense of distance. The neural representations of the terminals in the empty test interval, unrelated to the contour, could remain undisturbed, at least, less disturbed than the reference. Therefore, the representations of the two distances would not match. This would mean the expansion that was demonstrated in the experiments. It would be worthwhile to investigate the neural structures involved in performing size comparison tasks in the future. The literature data would provide support. The dorsal and ventral visual pathways depend on one another (Ayzenberg et al., 2023; Baizer et al., 1993; Gurariy et al., 2022; McIntosh & Schenk, 2009) and, according to an existing task, establish functional structures for representations (Lehky & Tanaka, 2016) of scenery, shape, position, grouping, and spatial interrelations. Hence, a functional network can be formed during a given experiment to compare the distances. Electrophysiological studies have provided insight into the integrity of this functional structure. In the human brain, alpha activity along the ventral visual pathway, including the left V_1_, proper LOC, and the bilateral inferior temporal cortex, is negatively correlated with object performance in size discrimination and illusion tasks (Chen et al., 2020).

In ordinary, everyday conditions, distortions in the perception of relative positions and distances often remain unnoticed but can be detected when performing a size comparison task. Most randomly chosen objects allow you to demonstrate the phenomenon of relative size expansion. An increase in the perceived distance can reach 20% or more of the length or height of a stimulus.

## Conclusions

We investigated the perceived expansion in size of the visual objects relative to empty space. The participants compared the distance determined by the two positions in the reference contour with the distance between the two test limiters. In most cases, the distance associated with the object contour is overestimated, as if the two positions are repelled from each other. The strength of overestimation varied depending on the shape of the stimuli. Among the geometric forms, rectangles were the most potent stimuli for increased relative size, circles and ellipses were weaker, and rhombuses were the least potent. Rectangles with different contours, such as lines, perceptual grouping, and spatial contrast in luminance, colour, and texture, caused approximately the same strength of overestimation. The illusory Kanizsa-type rectangles exhibited lower strength. The value of the overestimation gradually increased with the size of the stimuli for rectangles and triangles. The spatial effectiveness of the relative size expansion decreased exponentially.

In some instances, the lengths of the rhombus, triangle, and ellipse were underestimated. The structural components of the stimulus contour, such as angles and arcs, activate the lower neural mechanisms that encode the locations of parts of the stimulus contour using centroids. Shifts in a component's perceived location to the inside of the contour are considered contractions that weakened or exceeded the expansion. A balance of opposing neural processes determines the errors in estimating the relative size of stimuli. The stimuli were perceived as diminished when contraction was a dominant factor. The stimuli appeared longer and taller when expansion prevailed over contraction. The true magnitude of expansion was measured in the experiments with the Oppel–Kundt figure and low-ribbon-like stimuli owing to the absence of contraction.

The expansion can be interpreted as an inherent property of visual perception. The neural processing of an object's actual or subjective contours can serve as the primary inducer of expansion. Regularly arranged surface elements can strengthen the overestimation, while a homogeneous surface cannot. The expansion does not rely on the area or perimeter of the stimuli. This expansion mechanism may be related to the cortical areas of central vision. Associative units forming neural representations of contours at higher levels of the visual pathways could be considered candidates for the neural correlates of the size expansion.
